# Supplementary material for: Novel Tools to Measure Single Molecules Colocalization in Fluorescence Nanoscopy by Image Cross Correlation Spectroscopy
Source: Nanomaterials (Basel). 2022 Feb 18;12(4):686. doi: 10.3390/nano12040686 (PMC8875509; doi:10.3390/nano12040686)
Supplement: Supplementary file 1 [file nanomaterials-12-00686-s001.zip › nanomaterials-1559401-supplementary.pdf]

Supplementary materials

Novel Tools to Measure Single Molecules Colocalization in Fluorescence Nanoscopy by Image Cross Correlation Spectroscopy

Simone Pelicci [1], Laura Furia [1], Mirco Scanarini [1], Pier Giuseppe Pelicci [1,2], Luca Lanzanò [3,4] and Mario Faretta [1,*]

[1] Department of Experimental Oncology, IEO, European Institute of Oncology IRCCS, 20139 Milan, Italy; simone.pelicci@ieo.it (S.P.); laura.furia@ieo.it (L.F.); mirco.scanarini@ieo.it (M.S.); piergiuseppe.pelicci@ieo.it (P.G.P.)
[2] Department of Oncology and Hemato-Oncology, University of Milan, 20122 Milan, Italy
[3] Department of Physics and Astronomy "Ettore Majorana", University of Catania, 95123 Catania, Italy; luca.lanzano@unict.it
[4] Nanoscopy and NIC@IIT, CHT Erzelli, Istituto Italiano di Tecnologia, 16152 Genoa, Italy
* Correspondence: mario.faretta@ieo.it

Acquisition pipeline

Preliminary setup preparation

Timing: [varies]

1. Turn on the imaging setup.
2. Prepare fresh imaging medium (as described in Materials and Methods section).
3. Place a 125-µl drop of imaging medium onto the center of a 35mm Glass Bottom culture dish. Place one end of the coverslip on the dish and, with a tweezer, gently lower the coverslip onto the bottom of the dish so that the medium covers the entire section under the coverslip without forming any bubbles.
4. Position the specimen on the microscope and focus on a ROI with the objective used for 2color-STORM imaging (100x NA1.49 in our case). After turning on the lasers at minimal power (or maximum power with a Neutral Density filter), the immunolabeling should be visible in the, red and far-red channels according to the performed staining.

5. Align the laser incidence-angle to maximize the signal to noise ratio (generally with an inclined illumination close to the critical angle to minimize out-of-focus fluorescence contribution).
6. To check the blinking of the fluorophores, increase the power of excitation lasers used for 2-color dSTORM, to maximum; the sample fluorescence should disappear within seconds and spontaneous blinking of fluorophores be clearly visible. Upon brief pulses of illumination with low-power 405-nm laser, the density of blinking events should transiently increase.

dSTORM Imaging

Timing: [20 min]

Here, we described the single-molecule acquisition procedure for 2-color dSTORM.

7. Optimize the microscope parameters for STORM imaging for each channel. Turn on the imaging laser to a power of 70–90% and allow the fluorescent molecules in the sample to reach the dark state. Blinking should be visible at this stage. Set the camera parameters for optimal S/N ratio. Considering the density pattern of the staining and the duration of dye blinking events, it is generally suitable to use an exposure time of 20ms. Binning should be avoided.
8. Set the activation laser (405 nm) at minimal power and increase the power gradually until blinking becomes sufficiently dense. In our system, typically 1–3% of 405-nm laser power for Alexa Fluor-647 (or DyLight650) STORM imaging and 5–10% for the Cy3 (or CF568) can be used to optimize blinking of the dyes.
9. Set the activation mode and the number of frames. For dSTORM, we employed continuous activation mode (both the activator and imaging lasers are continuously on). The optimal number of frames per image depends on the type of experiment. In this work, we selected 15.000 frames per channel in continuous mode.
10. Activate a channel for drift correction. In our experiments the green channel (488nm) is employed to excite nanodiamonds fluorescence (40nm-Fluorescent Nanodiamonds (FNDs) conjugated to Streptavidin, Adamas Nanotechnologies). Set the acquisition interval of 488nm-channel during STORM imaging; we collected FNDs signal with a frequency of 1 every 1000 frames during the acquisition of each channel.
    Important: In each channel, every acquisition starts with a transitory phase to push all the molecules to the dark state. The duration of the transition depends on the employed dye, density of labelled molecules and power of the excitation and activation lasers.
11. Run the 2color-STORM real time acquisition on the two channels sequentially. During acquisition, adjust the activation laser power (405 nm) to optimize the number of blinking events.

Image reconstruction

Timing: [varies]

Here, we described the dSTORM image reconstruction of 2-color channels, aligned with 40nm-Fluorescent Nanodiamonds.

12. Open the software employed for detection of single molecule events (in our case NIS Elements Offline N-STORM Analysis module; several freeware software are available to perform molecule localization and reconstruction, such as ThunderSTORM, QuickPALM or RapidSTORM.) in STORM images.
13. Open the 647nm-channel STORM dataset and find the proper intensity parameters to identify single-molecule photon emissions
14. Select the minimum intensity to identify fluorescent Nanodiamonds peaks.
15. Activate the correction of the spatial drift between frames
16. Finally, run the analysis. Generally a reconstructed STORM image and a molecule list in a binary format are generated.
    Note: For 2D STORM experiments with high labeling density, it might be useful to discriminate the single events by deconvolving overlapping Gaussian PSFs. The aim of the analysis of the images acquired in a STORM experiment is the identification of blinking events and fitting a Gaussian function on each single-molecule image to determine the centroid position.
17. **IMPORTANT:** When using FNDs their localizations from the corresponding channel (488 nm excitation) are employed as reference point to calculate the drift correction. However FNDs are detected in all channels, given their spectroscopy properties. Localizations in their proximity should be consequently removed considering their physical size (40 nm) and the obtained localization precision.
18. Reconstruct the final STORM image by superimposing a Gaussian PSF to every localized molecule. The amplitude of the curve is proportional to the number of photons detected: the conversion factor (gray levels-photon number) is a parameter dependent on the employed camera generally provided by the producer. Choose the gaussian width (10 and 50nm in this work) of the single-localization PSFs and the format (10nm/pixel) of the reconstructed image.
19. Save the final STORM image
20. Repeat steps 14-20 for 561-channel STORM dataset.
21. Align the two Channels (647 nm and 561 nm) by the Nanodiamonds-peaks positions, calculated in the drift-correction channels.

Correlative Imaging

22. In correlative experiments, a confocal image is acquired before dSTORM imaging (step 7). After dSTORM acquisition and reconstruction (step 22), confocal and single-molecule images are aligned employing the FNDs as reference point for registration. The confocal image was rescaled to fit the size of dual-color STORM image. The detailed correlative procedure will be discussed in a future work (Pelicci et al., in preparation).

Computational pipeline

Images preparation procedure

Timing: [varies]

1. Open the STORM images of the 647 nm and 561 nm channels
2. Generate a mask of a region of interest (ROI) corresponding to the area of analysis (i.e. nucleus).
   Note: all the pixels outside this ROI were assigned an intensity value equal to the average value inside ROI. This step is useful to minimize the effects of nuclear borders on the correlation functions.
3. Save the single STORM images and mask image in .tif format.
   Note: all images must have the same format (i.e. 256x256, 512x512, etc) and same pixel-size.

ICCS Analysis

Timing: [varies]

4. Oneto et al.[1] provided an open-source code that was used in this work for ICCS analysis in MatLab. Download the user-friendly version of MATLAB script for ICCS analysis (https://github.com/llanzano/ICCS) and run the program.
5. Open files containing the images of first and second channel, respectively, and the mask image,
6. All images will be opened in "Figure 1" window. Set the parameters for ICCS analysis (Threshold, Smooth, Extra Pixels, Points to Plot for ACF) in the "Set area for analysis" window. Images will be processed for analysis on the basis of the active filtering and ROI settings.
   Note: Only pixels that are present within the selected ROI will be evaluated. If no ROI is selected, then all the pixels over the specified threshold will be used. If a mask image is loaded, filtering (threshold) is not required.
7. ICCS analysis script generates a graphical representation of the results. "Figure 2" window will show the auto-correlation curves of single-channels and the cross-correlation curve. The colocalized fraction f_{ICCS} and the distance d_{ICCS} values will be calculated and displayed. Use "Set parameters for fit" window for ICCS analysis (Min and Max lag, Tolerance, PixelSize, Full Width Half Maximum (FWHM)). Click OK button to update parameters and then click Cancel to continue.

   ICCS algorithm explanation:

   The analysis is based on the calculation of spatial autocorrelation (ACF) and cross-correlation (CCF) functions. As explained in Oneto et al.[1]:
   "The two-dimensional (2D) image correlation functions were calculated as:

$$G_{i,j}(\delta_x, \delta_y) = \frac{\langle I_i(x,y) I_j(x+\delta_x, y+\delta_y)\rangle}{\langle I_i(x,y)\rangle \langle I_j(x,y)\rangle} - 1$$

where the angle brackets indicate the average over all the pixels from the image, and $I_1(x,y)$ and $I_2(x,y)$ correspond to the images in the two-channels. To calculate the ACF we set i = j = 1 and i = j = 2. To calculate the CCF, we set i = 1 and j = 2. The numerator in the equation was calculated by a 2D fast Fourier transform algorithm. Next, the 2D correlation functions were converted into one-dimensional radial correlation functions, $G_{ij}(\delta r)$ by performing an angular mean as explained in Scipioni et al.[2]. The resulting radial correlation functions were then fitted to a Gaussian model

$$G_{ij}(\delta r) = G_\infty + G_{ij}(0)\exp(-\delta r^2/w_{ij}^2)$$

to extract the amplitude parameters $G_{ij}(0)$ and the width parameters w_{ij}. The width parameter corresponds to the $1/e^2$ of a Gaussian function and it is related to the full width half maximum (FWHM) by the relationship $w = FWHM/(2\ln 2)^{1/2}$. To calculate the co-localization coefficients M_1 and M_2 we used the amplitude parameter $M_1 = G_{12}(0)/G_{22}(0)$ and $M_2 = G_{12}(0)/G_{11}(0)$. To calculate the co-localized fraction f_{ICCS} we performed the arithmetic average of M_1 and M_2 coefficients. CCF was normalized to the ACF_1 (channel 1) and ACF_2 (Channel 2). The broadening of the cross-correlation function with respect to the autocorrelation functions was evaluated as $\Delta w = w_{12} - w_{cc}$ with $w_{cc} = ((w_{11}^2 + w_{22}^2)/2)^{1/2}$. The broadening of the CCF is indeed proved to be sensible only to the average distance separating the correlated particles. However, many experimental factors cause a broadening of the CCF such as background, particle aggregation and orientation, impacting both on the amplitude and width of the cross-correlation curve. The Δw value was converted into a distance value $d_{ICCS} = (\Delta w/C)^{1/2}$, where C is a constant determined from simulated data".

8. Save the results. The algorithm generates .txt and .mat files with auto- and cross-correlation fits data.
9. ICCS analysis script generates a graphical representation of the results. "Figure 3" window will show the local ICCS map, named "Local cross". Use "Parameter for local ICCS" window for local ICCS analysis and select the points for local ICCS maps.

Local ICCS explanation:

As reported in Oneto et al.[1], local cross correlation is iteratively calculated on small square 69 x 69 pixels wide subregions of the full-size image. For each subregion, the local ACF and CCF were calculated and the parameters $G_{ij}(0)$, w_{ij}, and f_{ICCS} were extracted as described for ICCS analysis. The resulting values were interpolated to produce a map of the same size of the full-size image.

10. Save the results. The algorithm generates .mat file and .tif image of spatial ICCS map.

References:

1. Oneto, M.; Scipioni, L.; Sarmento, M.J.; Cainero, I.; Pelicci, S.; Furia, L.; Pelicci, P.G.; Dellino, G.I.; Bianchini, P.; Faretta, M.; et al. Nanoscale Distribution of Nuclear Sites by Super-Resolved Image Cross-Correlation Spectroscopy. *Biophys. J.* **2019**, *117*, 2054–2065. doi:https://doi.org/10.1016/j.bpj.2019.10.036.
2. Scipioni, L.; Gratton, E.; Diaspro, A.; Lanzanò, L. Phasor Analysis of Local ICS Detects Heterogeneity in Size and Number of Intracellular Vesicles. *Biophys. J.* **2016**, *111*, 619–629. doi:https://doi.org/10.1016/j.bpj.2016.06.029.
